# A quasi-natural experimental study on enterprise innovation driven by urban agglomeration policies in China

**DOI:** 10.1038/s41598-023-37384-7

**Published:** 2023-06-26

**Authors:** Na Li, Saihu Song

**Affiliations:** 1 Faculty of Finance and Economics, Jimei University, No. 1, Jicen Road, Xiamen, Fujian People’s Republic of China; 2 The Research Center for Local Financial Performance, Jimei University, No. 1, Jicen Road, Xiamen, Fujian People’s Republic of China

**Keywords:** Socioeconomic scenarios, Sustainability

## Abstract

It is of great significance to carefully evaluate the actual impact of macro-policy formulation on promoting micro-enterprise innovation and implementing innovation-driven strategies. This study utilizes data from Chinese listed companies between 2012 and 2019 and takes the implementation of urban agglomeration policies as a natural experiment. By employing the multi-period differential method, the driving mechanism of urban agglomeration policies on enterprise innovation is investigated. The results show that: (1) Urban agglomeration policies effectively promote the enhancement of regional enterprises’ innovation capability. (2) Urban agglomeration policies reduce enterprise transaction costs through integration effects, mitigate the influence of geographical distance through spillover effects, and stimulate enterprise innovation. (3) Urban agglomeration policies have a regulatory effect on the siphon and spillover mechanism formed by the central city, thereby driving the innovation and development of peripheral micro-enterprises. (4) Further research from the perspectives of enterprises, industries, and locations reveals that the macro, medium, and micro effects of urban agglomeration policies differ, leading to heterogeneity in enterprise innovation responses. Therefore, it is necessary to continue promoting policy planning for urban agglomerations, enhance the coordination of urban policies within urban agglomerations, adjust the influence of the self-mechanism of urban agglomerations, and foster the formation of a multi-center innovation structure and network within urban agglomerations.

## Introduction

Amidst unprecedented global changes in the past century, the world economy and production structure have experienced significant transformations and restructuring. In this new landscape, a country’s competitive soft power is increasingly determined by its ability to foster innovation. Innovation has emerged as a crucial strategy for driving social and economic development, attracting widespread attention from governments. In China, innovation-driven development serves as a key strategic measure to construct a moderately prosperous society and achieve shared prosperity comprehensively.

Within the framework of innovation development, transformation, and implementation, the innovation activities of micro-market entities play a pivotal role. These activities are influenced by various factors such as regional innovation resource allocation, flow of regional factors, and innovation system methodologies. Moreover, regional disparities in development, institutional barriers, and the intensity of intra-regional connections all have an impact on micro-enterprise innovation. Therefore, conducting a careful evaluation of the actual impact of macro policy formulation holds great significance in promoting innovation within enterprises and implementing an innovation-driven strategy.

Numerous regional strategies have a significant impact on enterprise innovation, and the construction of urban agglomerations has emerged as a highly influential regional policy in recent years. Since the initiation of China’s reform and opening-up, the urbanization rate has steadily increased, surpassing 60% in 2020. However, the issue of resource allocation imbalance resulting from urban congestion remains prominent. In response, the “*Opinions of the CPC Central Committee and the State Council on Establishing a More Effective New Mechanism for Coordinated Regional Development*” in November 2018 explicitly outlined the promotion of integrated development within major national regional strategies, including the Beijing–Tianjin–Hebei urban agglomeration, the Yangtze River Delta urban agglomeration, the Guangdong–Hong Kong–Macao Greater Bay Area, the Chengdu-Chongqing urban agglomeration, the Yangtze River midstream urban agglomeration, the Central Plains urban agglomeration, the Guanzhong Plain urban agglomeration, and others. These urban agglomerations aim to establish a new model where central cities drive the development of urban agglomerations, and urban agglomerations drive regional development. The goal is to promote integrated and interactive development among regional clusters, leverage the advantages of concentrated distribution of cities and towns within urban agglomerations, benefit from economies of scale and scope, foster close division of labor and cooperation, facilitate factor resource flows, and enhance the efficiency of resource allocation.

Therefore, it is evident that the construction of urban agglomerations is conducive to breaking down intra-regional barriers and restrictions on factor flows. It removes obstacles that hinder the innovation and development of micro-entities and inevitably impacts the innovation and development of enterprises through external policies. Consequently, it is worth paying attention to several aspects. Firstly, whether the construction of urban agglomerations is committed to breaking down inter-regional barriers and obstacles within the region, enhancing cooperative advantages among cities and towns at all levels, promoting factor flows, and stimulating enterprise innovation. Secondly, as urban agglomerations represent larger spatial concentrations, their externalities primarily stem from the growth of surrounding hinterlands driven by the central city through knowledge innovation spillovers, reduction of transaction costs, industrial transfers, and other factors. It is essential to investigate whether urban agglomerations also exert spillover effects on enterprises and, if so, through which pathways. Thirdly, the potential siphoning effect of urban agglomerations may result in a hierarchical decrease in spillover effects, thus raising the question of whether enterprise innovation is also influenced by the attenuation of geographical distance.

Existing studies have primarily focused on the macro perspective when examining the impact of urban agglomeration on regional economic development. It is widely acknowledged that urbanization can stimulate regional economic growth^1–6^ and facilitate the upgrading of regional industrial structures^7,8^. Additionally, urban agglomerations can enhance regional innovation capabilities through positive spillover effects^9–11^. However, urban agglomeration also brings about a series of social and environmental challenges such as regional development gaps^12,13^ and air pollution^14–17^.

Nonetheless, urban agglomerations have the ability to allocate and integrate resources on a broader scale, facilitating regional economic development by transferring industries to smaller cities^18^. This approach helps alleviate problems stemming from excessive concentration in larger cities, promotes regional economic growth, and enhances the coupling and coordination between urbanization and innovation. However, economic growth exhibits significant spatial variations among individual cities within the urban agglomeration^19,20^.

Based on these observations, scholars have investigated the relationship between urban agglomeration and enterprise innovation and growth from the perspective of micro-enterprises. Zhao et al*.*^21^ analyzed the impact of establishing urban agglomerations on innovation by utilizing data from Chinese listed companies and employing quasi-natural experiment methods. They found that the establishment of national-level urban agglomerations encouraged enterprises to increase R&D expenditure and enhances their innovation output. Another study by Zhao et al*.*^22^ focused on the Yangtze River Delta and the Pearl River Delta, demonstrating that the establishment of urban agglomerations in these areas can moderately improve the innovation capabilities of enterprises. Their quantitative analysis revealed that financial support and regional coordination played crucial roles in promoting enterprise innovation^22^. Chen et al.^23^, in their research on Asian cities and enterprises, suggested that enterprises in larger cities are more motivated to introduce product and process innovations, indicating that urban agglomeration fosters enterprise innovation. Furthermore, they found that the accumulation of human capital was a key driver behind the promotion of enterprise innovation within urban agglomerations^23^.

However, existing research on urban agglomerations and micro entities primarily focuses on the patterns and paths of enterprise innovation within specific urban agglomerations. There is limited attention given to the effects of urban agglomeration policies on enterprise innovation and their underlying mechanisms. This knowledge gap may impede performance research and hinder accurate assessment of macro policy formulation, thus hindering precise planning for subsequent policy implementation. Therefore, this study aims to investigate the spatial effects and driving mechanisms of urban agglomeration policies on enterprise innovation, using a quasi-natural experiment approach to provide scientific evidence for the precise implementation of urban agglomeration policies and urban management planning. In contrast to previous studies, this paper’s potential marginal contributions are as follows: Firstly, it integrates the analysis framework of macro policy implementation in urban agglomerations with the responses of micro-market players, expanding the discussion on the spatial spillover effects of macro policy implementation. It sheds light on the impact mechanisms of major regional policies on micro-enterprise innovation and their differential effects across heterogeneous regions, thereby enriching the research field of innovation economic geography. Secondly, this study employs the quasi-natural experiment method to simulate the integration effect of policy implementation and the heterogeneous effects of geographic distance attenuation. By conducting mechanism testing, it explores the impact of urban agglomeration policies on enterprise innovation and enhances our understanding of the driving mechanisms of regional macro policies on enterprise innovation. This approach enables a more comprehensive examination of the effects of urban agglomeration policies and provides insights into the nuanced dynamics between macro policies and micro-level innovation processes. Thirdly, this study delves into the enterprise innovation response triggered by urban agglomeration and examines the spatial variations in the impacts of urban agglomeration policies across different scales, taking into account factors such as property rights, industry characteristics, and location heterogeneity. By doing so, it offers valuable insights for exploring precise planning and continuous implementation of macro regional policies. This analysis provides a nuanced understanding of how various factors interact within different contexts, offering guidance for policymakers seeking to optimize the outcomes of urban agglomeration policies.

## Mechanism analysis and research hypothesis

New economic geography believes that the distribution of firms in space tends to agglomerate due to the cost of icebergs. There are positive externalities and negative externalities in agglomeration, positive externalities can promote enterprise agglomeration, and negative externalities have an inhibitory effect on agglomeration. The “center-periphery” theory holds that whether economic activity is agglomerated or decentralized in space depends on who dominates the centripetal force (economies of scale, etc.) that concentrate industrial geography and the centrifugal force (ground rent, etc.) that concentrates industrial geography. Under the combined action of these two forces, the agglomeration and dispersion of enterprises in geographical space will continue to adjust, and the allocation of resources and the flow of factors will also change. This provides a theoretical basis for us to analyze the flow of factors and the spatial distribution of industries in urban agglomerations.

**Figure 1:**
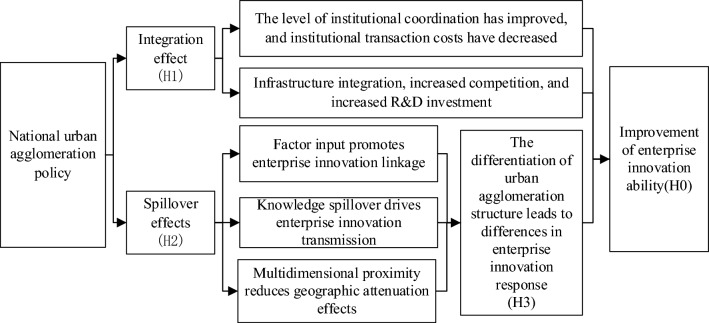
Mechanism analysis.

Different from the development model of individual cities, the development plan of urban agglomeration contains the development direction, goals and realization paths of the urban agglomeration as a whole, as well as the positioning, development goals and direction of each city in the urban agglomeration. After the establishment of urban agglomerations, cities will, in accordance with the requirements of urban agglomeration development planning, strengthen the interconnection of infrastructure between cities, reduce institutional and policy barriers, promote the optimal allocation of resources on a larger scale, and improve the efficiency of resource allocation. According to its own comparative advantages, each city will promote industrial transfer within the urban agglomeration in an orderly manner, adjust the industrial layout, optimize the industrial division of labor, and improve the level of industrial coordination between cities. The optimization and adjustment of industrial layout within urban agglomerations can effectively reduce the problem of industrial homogeneous competition between cities, improve the level of specialized and diversified agglomeration of industries, give full play to the positive externalities of agglomeration, and improve the innovation performance of enterprises. Therefore, the implementation of urban agglomeration policy can improve the innovation performance of enterprises by improving the efficiency of resource allocation and giving play to the positive externalities of agglomeration (Fig. 1).

### Urban agglomeration, integration and corporate innovation

Industrial agglomeration is a large-scale production organization formed by local micro market entities in specific regions. It generates economies of scale and scope, reduces production costs, and improves marginal returns of enterprises through the exertion of Marshall effects such as labor pool, intermediate goods input, and knowledge technology spillover^24^. In the context of scientific and technological innovation gradually replacing the traditional factors to become the driving force of economic growth^25^, the improvement of enterprise cost–benefit ratio is conducive to promoting enterprise input innovation, generating avoidance of competition and expanding innovation output. The reduction of production costs can be achieved through two paths: knowledge spillover and network transmission. In terms of knowledge spillover, an informal information communication platform such as technology and market is formed to promote the transmission of “sticky knowledge” through the concentration of the same industry and intermediate products input industry; on the other hand, the reservoir generates a gravitational effect to avoid the exclusive constraints of human capital investment, promote the human capital spillover effect, and reserve innovative talents, through leading enterprises to drive talent gathering. In terms of network transmission, the agglomeration of the same industry and intermediate products industry is easy to form a specialized and diversified division of labor, reduce the transaction costs of members in the agglomeration, and form a vertical complementary system of cooperation advantages. With this system, enterprises can share “sticky knowledge”, innovate processes, reduce costs, increase innovation output, and produce positive externalities in knowledge technology, production skills and production links^25^. However, when clusters become overcrowded, for example, with the increase of clustered enterprises, land rent, wages, environmental pressure and traffic congestion are increasing, leading to the increase of the marginal cost of clustered enterprises, and the negative externality effect appears, which will greatly reduce the increase in returns to scale brought about by clustering, and even promote the decentralization of clustering. At this time, if enterprise innovation cannot produce the behavior of avoiding competition, it is hard to avoid falling into the Schumpeter effect, reducing the innovation profits, and the enterprises also lack the motivation for innovation. Therefore, the positive effect of agglomeration can promote enterprise innovation by reducing production costs, while the negative effect can inhibit enterprise innovation by increasing production costs.

Urban agglomeration seems to represent the geographic spatial agglomeration in a larger scope^19^, the agglomeration scale is further expanded through urban agglomeration policies, thus exerting agglomeration effect in a larger scope and influencing the innovation of enterprises. Existing theoretical and empirical studies have revealed that urbanization promoted regional innovation capability through positive spillover effects^10,11^, and also pointed out social and environmental problems brought about by urbanization^12–17^. The causes of urban diseases are as the same as those of agglomeration and dispersion, and the solutions are similar, which tend to upgrade the internal industry, restructure the functions and spread the external hinterland to achieve the goal of a virtuous circle and sustainable development. The difference is that there are many administrative planning means available in the process of urban disease treatment, and whether the agglomeration can get rid of the decentralized force mainly depends on the stable upgrading of the collaboration relationship between the micro market players, but no matter which one needs to reach a relative equilibrium state through market regulation ultimately. The solution of urban disease problems will also lead to the solution of the contradiction of agglomeration congestion, and the driving role of innovation is particularly critical.

The introduction of urban agglomeration policies is conducive to promoting the governance of urban diseases, the generation of innovation and the healthy development of regional economy^26–30^. Ding et al.^19^ used DID method to conduct an empirical analysis of seven national urban agglomerations, and believed that national urban agglomerations can effectively drive regional economic growth, and the driving effect was affected by spatial distance and the economic development level of central cities. Li et al.^20^ took the implementation of China’s regional integration policy as a quasi-natural experiment and found that regional integration policy can effectively promote the level of regional green innovation. Tan et al.^31^ analyzed data on urban agglomerations in China and found that the improvement of regional integration can promote the development of green innovation, mainly through the promotion of foreign direct investment, economic agglomeration and financial development. The study of Shen et al*.*^32^ found that the development model of urban agglomeration can effectively improve the carrying capacity of land resources and help to improve the economic performance of cities within urban agglomerations. Therefore, the urban agglomeration policies may have an impact on a larger range of spatial agglomeration^33^, and then produce policy spillovers to micro-market entities, that is to promote the reduction of enterprise transaction costs by means of the integration effect of urban agglomerations: firstly, the urban agglomerations improve the level of integrated markets, Ren et al*.*^34^ believed that cities within urban agglomerations mainly promoted the improvement of the degree of integration of the urban agglomerations through the interconnection of transportation infrastructure and the diversification of economic connections. Secondly, the city cluster should improve the level of integration system. With the city governing counties, the merger of districts and the city alliance as the main governance model^35^, under the city alliance system, the inter-regional consultation and cooperation mechanism is more perfect. Administrative barriers and institutional barriers between cities within the city cluster are gradually weakened or eliminated through top-level design such as regional development integration, and regional policy coordination is enhanced. The government service *“All in One Network”* has realized the unification of regional rules and regulations and the convenient sharing of public services, greatly reducing the time for administrative approval, driving down the institutional transaction costs of enterprises, enabling enterprises to invest more in production and research and development, which is conducive to the improvement of enterprise innovation capability^36^. Thirdly, the urban agglomeration will improve the level of integrated infrastructure. The construction of urban agglomeration will continue to improve the level of transportation infrastructure integration, further promote the reduction of regional segmentation and market transaction costs, and gradually form a unified regional market. In order to obtain more market share and profits, enterprises will actively explore new markets and face competition from more peer enterprises. Under the competitive effect, in order to maintain and expand their technological advantages, enterprises will increase the intensity of R&D investment and improve their technological level.

### Urban agglomerations, spillover effects and corporate innovation

In addition to the research on agglomeration and innovation, the New Economic Geography School further pays attention to the impact of spatial differences and geographic distance attenuation on urban agglomeration effects^37^. Similar to agglomeration effects, urban agglomeration also encompasses both positive and negative externalities. The magnitude of spillover effect mainly depends on the connectivity of the transportation infrastructure between the city and the surrounding cities^38^. He et al.^39^ found that central cities played an important role in the regional development through research on China’s Chang Zhu Tan urban agglomeration, and central cities promote the development of surrounding areas through spillover effects. Zheng and Du^40^ found that central cities had siphon effect on surrounding areas based on their better economic development level and better traffic connectivity through the analysis of data of nearly 300 prefecture level cities in China. Sun et al*.*^41^ believed that urban agglomeration policies had a more significant effect on the economy of central cities, and even had a negative effect on non-central cities, that was, there was a shadow of agglomeration. Kang et al*.*^42^ believed that when the level of regional integration was low, the regional gap could be improved, and when the level of regional integration was high, the regional gap will be expanded. The stronger the economic strength of a central city is, the closer the geographical distance between it and its surrounding cities is, the larger the scope of spillover and siphon effects will be. The borrowing scale and gathering shadow generated by surrounding cities relying on large cities will decline with geographical distance. Structural differences caused by spatial differences lead to different spillover effects of urban agglomerations in different regions, and geographical distance is the key factor affecting the size of spillover effects in the same urban agglomeration. However, it should be emphasized that the overarching objective of implementing urban agglomeration planning policies is to achieve balanced development through the collaborative efforts of cities and towns, leveraging their respective advantageous industries. At different stages of development, the country will undertake distinct policy planning strategies. The promotion of urban agglomeration policies can bring benefits to a larger number of cities and towns, potentially reducing the extent of geographical attenuation.

The geographical difference of spillover effect of urban agglomeration surrounding areas may also have a linkage effect on the innovation of micro market players: firstly, due to the existence of information asymmetry and incomplete information, enterprises in the same urban agglomeration will have an imitation effect^43^. The innovation behavior of enterprises in urban agglomeration also has a linkage effect, which can be achieved through capital investment and labor investment. Therefore, if the spillover effect of urban agglomeration is dominant, it will increase the capital and labor input of surrounding cities and stimulate the enterprise innovation of surrounding cities. If the siphon effect of urban agglomeration is obvious, the central city will further attract the capital and labor of surrounding cities and have an impact on the enterprise innovation of surrounding cities. Secondly, knowledge spillover is an important driving force for enterprise innovation under the spatial agglomeration of urban agglomerations. For urban agglomerations dominated by spillover effect, the viscous knowledge is conducive to technology exchange, production link transmission and intermediary communication through the integrated market, which is conducive to innovation of enterprises in surrounding cities. On the contrary, urban agglomerations dominated by siphon effect are more likely to reflect the polarization of enterprise innovation. Thirdly, many studies have pointed out that the spillover of knowledge and technology was not limited to the region, and multi-dimensional proximity such as geography, knowledge, society, culture and transportation would promote innovation activities between regions^44^. The small cities in urban agglomerations can borrow the externalities of large-scale urban agglomeration^45^ to form a trans-regional network transmission mechanism. Therefore, the urban agglomerations with large spillovers can further build regional network platforms through integration, promote the network transmission of innovative knowledge, and promote the innovative development of enterprises. The deduction from the existing research shows that: due to the structural differences of spillover effects in different urban agglomerations, the innovation spillover effects on enterprises are heterogeneous. Whatever the siphon effect or the spillover effect, the spillover effect of urban agglomeration on enterprise innovation is still dominated by proximity under the condition that the underlying logic of the existing industrial infrastructure construction production network remains unchanged. However, the goal of the implementation of the urban agglomeration policy planning is to minimize the impact of geographical attenuation, so as to achieve the balance of urban and urban development. Therefore, with the deepening of the implementation of the urban agglomeration policy planning, the spillover scope in the urban agglomeration will increase.

Based on the above analysis, this paper puts forward the following assumptions about the micro mechanism of the urban agglomeration policy on enterprise innovation and makes an empirical test on them later:

#### Hypothesis $\mathrm{H}0$

Urban agglomeration policy implementation can promote the improvement of enterprise innovation ability.

#### Hypothesis $\mathrm{H}1$

The implementation of urban agglomeration policy reduces the production cost of enterprises and promotes the enterprise innovation through integration effect.

#### Hypothesis $\mathrm{H}2$

The spillover effect of urban agglomeration policy on the promotion of enterprise innovation is affected by the attenuation of geographical distance, however the impact will gradually weaken with the implementation of urban agglomeration policy.

#### Hypothesis $\mathrm{H}3$

The structural differentiation of urban agglomerations results in varying degrees of impact on the innovation and development of enterprises. Moreover, heterogeneous enterprises exhibit different innovation responses to urban agglomeration policies.

## Empirical analysis and robustness test

### Model design and variable processing

Urban agglomeration planning is a comprehensive regional development plan formulated jointly by the governments of cities that have the potential to form an urban agglomeration based on their existing urban development situation. Once approved by the State Council of China, it becomes a national urban agglomeration. The purpose of urban agglomeration planning is to promote coordinated and sustainable development in the region, taking into account the overall regional perspective. For example, in 2016, the executive meeting of the State Council of China adopted the Yangtze River Delta City Cluster Development Plan and the Chengdu–Chongqing City Cluster Development Plan, marking that the Yangtze River Delta City Cluster and the Chengdu–Chongqing City Cluster have become national urban agglomerations and started the construction of urban agglomerations. The implementation of urban agglomeration policies signifies a new stage in the development of urban agglomerations. By considering urban agglomeration policies as a significant variable, a quasi-natural experiments can be conducted to examine the developmental effects of urban agglomerations. This approach allows it to analyze and evaluate the impact and outcomes of urban agglomeration policies on the overall development of urban agglomerations.

In order to avoid the endogenous problem between spatial agglomeration and enterprise development as much as possible, this paper uses the practice of the resent research for reference^46,47^, introduces the exogenous variable of urban agglomeration policy, and examines the impact of urban agglomeration on enterprise innovation and development. There are 11 state-level urban agglomerations officially approved by the State Council in 2021, and the policy time span is from 2015 to 2018. Due to the different time of policy promulgation, this paper adopts the multi-period DID method to establish an empirical model. In consideration of the availability of data and the formation time of urban agglomeration, referring to the practice of Ding et al*.*^19^, Guangdong Hong Kong Macao Greater Bay Area is selected by its predecessor Pearl River Delta urban agglomeration as the experimental group, and the policy time is adjusted from 2018 to 2015. Then the samples are divided into the experimental group and the control group. The cities of the experimental group after the implementation of the policy are assigned by a value of 1, and the cities of the control group without the implementation of the policy and the experimental group without the implementation of the policy are assigned by a value of 0. And then construct the benchmark regression model of this paper as follows:

1 $$innovate_{it} = \beta_{0} + \beta_{1} DID_{it} + \beta_{2} X_{it} + \mu_{r} + \lambda_{t} + \varepsilon_{it}$$

In model (1), ${innovate}_{it}$ represents the level of innovation of city i in the t period; ${\beta }_{0}$ is the intercept term; ${DID}_{it}$ is a binary dummy variable due to the difference in the implementation time of individual policies, if city i joins the city agglomeration in year_t_, the city is assigned by a value of 1 in year_t_ and subsequent years, otherwise the assignment is 0, and its coefficient, ${\beta }_{1}$ is the focus of this paper; ${X}_{it}$ represents control variables, including enterprise size, enterprise age, asset-liability ratio, government subsidies, growth capacity, return on assets, cash flow, etc. (Table 1); ${\lambda }_{t}$ represents a time-fixed effect; ${\mu }_{r}$ indicates an industry fixed effect; and ${\varepsilon }_{it}$ indicates a random perturbation term.

**Table 1:** Definition and Calculation of Main Variables.

Variable	Variable symbol	Variable construction
Enterprise innovation level	$innovate1$	Take the natural logarithm of the number of enterprise patents added by 1
	$innovate2$	Take the natural logarithm of the number of enterprise invention patents added by 1
Enterprise scale	$size$	Take the natural logarithm of the number of employees
Enterprise age	$age$	Take the natural logarithm of the establishment time of the enterprise
Asset liability ratio	$lev$	(Total liabilities/total assets) * 100
Government subsidies	$sub$	Natural logarithm of government subsidy amount
Growth ability	$growth$	(Current operating income—previous operating income)/previous operating income
Return on assets	$roa$	Net profit/total assets
cash flow	$cashflow$	Net cash flow from operating activities/total assets

The data time range is selected from 2012 to 2019, the data of A-share listed companies in China are selected as the research sample, and according to the convention, the financial, $ST$, $*ST$ and samples with serious variable defects are excluded, and finally 16,160 pieces of data of 2020 listed companies are obtained.

Among them, the enterprise patent data comes from the $Wind$ database, and the other enterprise data comes from the $CSMAR$ database. In view of the fact that it generally takes more than one year from application to approval, in order to reflect the changes in the innovation ability of listed companies more accurately, enterprise patents and enterprise invention patents are treated with a lag of one period. To eliminate the influence of extreme values on the results, a $Winsorize$ tail shrinkage of 1% is used for the main continuous variables. Table 2 shows descriptive statistics for the main variables.

**Table 2:** Descriptive statistics of main variables.

Variable	Obs	Mean	Min	Max	S.D
$innovate1$	16,160	3.148	0	11.025	2.34
$innovate2$	16,160	1.994	0	10.148	1.849
$size$	16,160	7.825	4.615	11.252	1.266
$age$	16,160	2.813	1.609	3.434	0.363
$lev$	16,160	44.863	5.518	92.679	21.019
$sub$	16,160	18.226	14.782	22.896	1.604
$growth$	16,160	0.451	− 0.679	9.773	1.295
$roa$	16,160	0.048	− 0.244	0.231	0.063
$cashflow$	16,160	0.088	− 0.684	0.716	0.187

From the descriptive statistics of the main variables in Table 2, it can be seen that the minimum value of innovate1 is 0, the maximum value reaches 11.025, the average value is 3.148, and the standard deviation reaches 2.34, indicating that the selected sample enterprises have large differences in innovation ability. The sample data of Innovate2 also exhibit this feature, with a minimum value of 0, a maximum value of 10.148, a mean of 1.994, and a standard deviation of 1.849. In addition, other control variables also differ greatly between sample companies, which provides a rich data basis for empirical research.

### Benchmark model regression

Table 3 reports the results of multi-phase DID regression, model 1 does not add control variables, model 2 adds control variables. In the case of controlling industry effect and time effect, without adding the control variable, the coefficient, ${\beta }_{1}$ is equal to 0.189, which is positive and passes the significant level of 5%. After adding the control variable, the coefficient, ${\beta }_{1}$ numerical value and significance decreased, but remained positive and passed the significant level of 10%. The results of the two returns show that the policy of urban agglomeration can promote the improvement of enterprises’ innovation ability. Hypothesis H0 is verified. The third model presents the regression results with the logarithm of enterprise patent applications plus one as the dependent variable. The regression coefficient of 0.207 is statistically significant at the 1% level, indicating that the establishment of national urban agglomerations has a positive impact on the number of patents applied for by enterprises and the number of patents authorized.In Model 4, the dependent variable is the logarithm of enterprise R&D investment plus one. The regression coefficient is 0.651, which is also statistically significant at the 1% level. This suggests that the establishment of national urban agglomerations has a positive influence on enterprise R&D investment.After replacing the quantitative index of enterprise innovation level, the return result is still significant, indicating that the establishment of national urban agglomerations can indeed promote the improvement of enterprise innovation ability.

**Table 3:** Benchmark regression results.

Variable	Model I	Model II	Model III	Model IV
DID	0.189**	0.141*	0.207***	0.651***
	(2.21)	(1.78)	(3.91)	(3.35)
Size		0.447***	0.554***	1.431***
		(12.00)	(21.74)	(13.33)
Age		− 0.783***	− 0.379***	− 2.321***
		(− 6.54)	(− 5.05)	(− 8.27)
Lev		− 0.00827***	− 0.00408***	− 0.0288***
		(− 4.08)	(− 3.14)	(− 5.16)
Sub		− 0.0266	0.142***	0.0595
		(− 1.00)	(7.90)	(0.75)
Growth		− 0.00510	0.00983	− 0.0749
		(− 0.33)	(0.85)	(− 1.36)
Roa		0.809*	1.387***	0.305
		(1.94)	(5.18)	(0.27)
Cashflow		0.0573	− 0.172**	− 0.163
		(0.50)	(− 2.00)	(− 0.41)
_cons	3.068***	2.604***	− 3.141***	9.730***
	(56.94)	(4.56)	(− 8.87)	(7.00)
Induetry	Yes	Yes	Yes	Yes
Year	Yes	Yes	Yes	Yes
N	16,160	16,160	16,160	16,160
r2	0.391	0.450	0.519	0.491

* * *, * * and * represent t $t$ he significance levels of 1%, 5% and 10% respectively, and the values in brackets are the values of statistics. The same below.

### Robustness test

#### Parallel trend test

**Figure 2:**
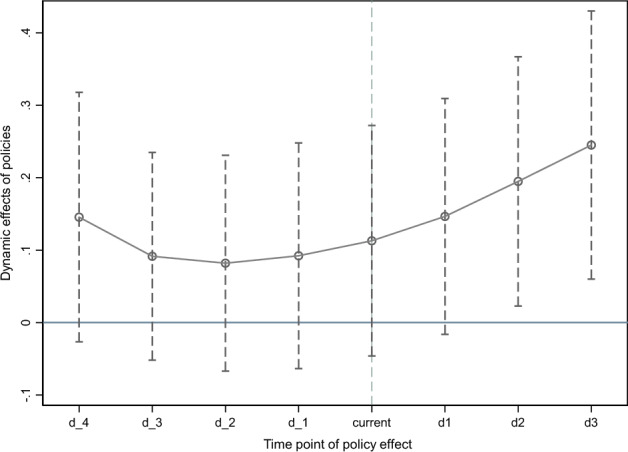
Results of parallel trend test.

When using the double difference method to evaluate the policy effect, in addition to the requirement that the area where the policy is implemented is completely random, a basic assumption that needs to be met is that there is no systematic significant difference between the experimental group and the control group before the implementation of the policy, that is, the experimental group and the control group have the same change trend before the implementation of the policy. In order to ensure the reliability of the research conclusion of the benchmark multi-period DID model, it is necessary to conduct parallel trend test on two groups of samples. This paper tests the robustness of the first four periods and the last three periods at the time point of policy implementation. The test results are shown in Fig. 2. It can be seen from Fig. 2 that the regression results of coefficients in each period before the policy implementation are not significant. It shows that there is no systematic difference between the treatment group and the control group before the implementation of the policy, and the two groups of samples have the same parallel trend, so the parallel trend test is passed.

#### Eliminate central cities

The DID method can well solve the endogenous problem through the intra group and inter group difference, and is widely used in policy effect evaluation. In this paper, although the dual difference method can well avoid endogenous problems, there is still the possibility of endogenous. To evaluate the effect of the policy, we need to assume that the policy is completely exogenous, that is, the choice of the treatment group and the control group is completely random. However, the establishment of national urban agglomeration is obviously not random. From the establishment of each urban agglomeration, we can see that each urban agglomeration has one or more central cities with good development level, and the central city is taken as the center to expand to surrounding cities. Therefore, there is a possibility that the city itself has a high level of development, and enterprises set up in the central city in order to obtain the positive externalities of the central city when selecting the site. The high rent and high labor costs in the central cities will “crowd out” enterprises with poor development from central cities, leaving the enterprises with strong strength and strong innovation ability. The development level of the central cities is relatively high, while the peripheral cities of the urban agglomeration are numerous, and most of them are not at a high level of development, and some will even spread out of the province. For example, the Central Plains urban agglomeration includes not only most prefecture level cities in Henan, but also some cities in Shandong and Shanxi. If the central cities within the urban agglomeration are removed from the regression sample and only the surrounding cities are investigated, there will be no problem of the enterprises’ self-selection. At the same time, it can also be explained that the policy effect after the establishment of the urban agglomeration has affected the improvement of the innovation ability of enterprises in the peripheral cities. Therefore, this paper uses the method of deleting the sample of central cities to solve the endogenous problem.

The regression results after excluding the central cities are shown in Table 4. As can be seen from Table 4, the coefficient ${\beta }_{1}$ equals 0.183 and passes the significant level of 10%. After removing the central cities and only performing regression on the samples of peripheral cities to solve the endogenous problem, the sample regression results are still significantly positive, indicating that the research conclusions of this paper are reliable.

**Table 4:** Robustness test results.

Variable	Eliminate central cities	Replace dependent variable	One installment in advance
DID	0.183*	0.165**	
	(1.89)	(2.47)	
Advanced_ did			0.130
			(1.60)
Size	0.550***	0.364***	0.448***
	(10.87)	(12.16)	(12.01)
Age	− 0.672***	− 0.519***	− 0.783***
	(− 4.51)	(− 5.33)	(− 6.54)
Lev	− 0.00747***	− 0.00485***	− 0.00826***
	(− 2.82)	(− 3.09)	(− 4.08)
Sub	− 0.0793**	0.0126	− 0.0268
	(− 2.33)	(0.58)	(− 1.01)
Growth	0.00246	0.00452	− 0.00476
	(0.12)	(0.38)	(− 0.31)
Roa	0.425	0.704**	0.807*
	(0.85)	(2.13)	(1.93)
Cashflow	0.224	0.0733	0.0564
	(1.36)	(0.86)	(0.49)
_ cons	2.596***	0.478	2.596***
	(3.60)	(0.97)	(4.53)
Industry	Yes	Yes	Yes
Year	Yes	Yes	Yes
N	8880	16,160	16,160
r2	0.443	0.396	0.450

The deleted central cities include Beijing, Wuhan, Changsha, Nanchang, Guangzhou, Shenzhen, Chengdu, Chongqing, Shanghai, Nanjing, Hangzhou, Zhengzhou, Harbin, Changchun, Nanning, Xi'an, Hohhot, Lanzhou and Xining.

#### Replace the dependent variable

This paper uses the enterprise patent data plus one logarithm as the proxy variable of enterprise innovation capability in the benchmark regression. The patent data includes invention patents, utility model patents and design patents, among which invention patents are the most important of the three patents. The application for invention patent needs to pass a series of strict examinations, especially in the aspects of novelty, creativity and practicality, which can better reflect the scientific and technological strength and innovation ability of enterprises. Therefore, this paper selects the number of invention patents as the dependent variable for robustness test. The regression results of replacement dependent variables are shown in Table 4. It can be seen from Table 4 that the coefficient regression result is 0.165, which is significant at the 5% significance level. Using the invention patents to replace the number of enterprise patents for empirical regression, the results are still significantly positive, indicating that urban agglomeration policies can indeed promote the improvement of enterprise innovation capability in urban agglomeration.

#### Counterfactual simulation

In the application process of the double difference method, it is necessary to verify that the significant difference in the development trend between the experimental group and the control group really occurs after the implementation of the policy. Therefore, the implementation time of each phase of the policy will be advanced uniformly by one year. For example, the transit time of the urban agglomeration established in 2015 will be adjusted to 2014, and so on. Regression is conducted according to the adjusted policy implementation time, and the regression results are shown in Table 4. The regression coefficient is 0.13, which does not pass the 10% significant level. Therefore, the counterfactual simulation is passed, and the conclusion from the benchmark DID is still stable, that is, the urban agglomeration policy promotes enterprise innovation.

### Endogenous problems

The double difference method can effectively alleviate the endogenous problem through two differences, but there may still be the following endogenous problems: (1) There may be a two-way causal relationship between the implementation of urban agglomeration policies and enterprise innovation. Urban agglomerations are often urban clusters formed and developed around one or more cities with high economic development level and strong innovation ability, and the development level of this urban agglomeration is higher than that of other cities. Urban agglomeration policies are often first implemented in urban agglomerations with high economic development levels and strong innovation capabilities. That is, due to the strong innovation ability of enterprises in the city, the urban agglomeration policy is implemented in the city. (2) Non-observational factors and non-complete randomization of samples in the experimental group may lead to endogenous problems. Therefore, this paper chooses the tool variable method to control possible endogenous problems.

Therefore, the distance (Di) between each city and the central city is selected as the instrumental variable for the following reasons: (1) Correlation, the implementation scope of the urban agglomeration policy is closely related to the distance from the central city. (2) Exogenous, the exogenous characteristics between the distance from the central city and the level of enterprise innovation are more significant.

Then forward the instrumental variables: subtract the distance from the central city from the central city, take the absolute value, and finally divide by 100. After that, the 2SLS model is used for testing. Table 5 reports the regression results based on the instrumental variable method. The regression results in the first column reflect the correlation between the core explanatory variables and the instrumental variables, and the results show a significant positive relationship at the significance level of 1%, indicating that there is a positive relationship between the distance from the central city and the implementation of the urban agglomeration policy, indicating that the selection of instrumental variables is valid. The F statistic value of the weak instrumental variable test is 477.571, which is greater than the critical value of 10, indicating that there is no obvious weak instrumental variable problem. The regression results of the second stage reflect the consistent estimator of the structural parameters obtained by the instrumental variable method, and the results are significantly positive. The results of this model show that after alleviating endogenous problems such as bidirectional causation, urban agglomeration policies still have a promoting effect on the improvement of enterprises’ innovation ability.

**Table 5:** Regression results of instrumental variables.

Variables	First	Second
	DID	Innovate1
Di	0.056***	
	(70.436)	
DID		0.935***
		(20.333)
Size	0.000	0.711***
	(0.111)	(42.659)
Age	− 0.021***	− 1.199***
	(− 2.772)	(− 23.852)
Lev	0.000	− 0.017***
	(1.419)	(− 17.420)
Sub	0.002	− 0.219***
	(1.215)	(− 15.987)
Growth	− 0.000	− 0.060***
	(− 0.045)	(− 4.483)
Roa	0.057	− 0.834***
	(1.389)	(− 2.861)
Cashflow	− 0.033**	− 0.663***
	(− 2.461)	(− 7.090)
Constant	− 1.120***	5.433***
	(− 25.946)	(22.754)
Induetry	Yes	Yes
Year	Yes	Yes
Observations	16,160	16,160
R-squared	0.644	0.162

## Mechanism inspection

### Integration effect

The construction of urban agglomerations can improve the integration level of market, system and infrastructure, improve market capacity, reduce enterprise transaction costs, stimulate enterprises to participate in market competition in a larger scope, and improve the motivation of enterprises to invest in innovation. Therefore, this paper draws on the practice of Wang and Feng^36^, uses the ratio of the sum of enterprise sales expenses, management expenses and financial expenses to total profits to measure institutional transaction costs, multiplies with the double difference term (DID), and then regresses with the dependent variable. At the same time, considering that it is also necessary to measure whether the driving force of enterprise R&D investment comes from the integration effect of urban agglomeration, this paper continues to test the integrated adjustment effect by taking the logarithmic representation of the R&D intensity of the enterprise and the double difference term (DID) multiplication of the R&D investment of the enterprise, and then regressing with the dependent variable.

The mechanism test model is as follows:

2 $$innovation_{it} = \beta_{0} + \beta_{1} DID_{it} *N_{it} + \beta_{2} DID_{it} + \beta_{3} N_{it} + \beta_{4} Z_{it} + \lambda_{t} + \mu_{i} + \varepsilon_{it}$$

Among them, ${innovation}_{it}$ represents the level of innovation of enterprise i in the t period, ${DID}_{it}$*${N}_{it}$ represents the multiplication term of the regulatory variable and the DID term, ${DID}_{it}$ is the binary dummy variable due to the difference in the implementation time of individual policies, ${N}_{it}$ is the regulating variable, and ${\beta }_{1}$ is the core explanatory variable. The other variable settings are consistent with the baseline regression model.

Table 6 reports the regression results of the impact of the integration effect, column 1 is the institutional transaction cost regression result, and did1 represents the multiplication of the institutional transaction cost and DID term. Column 2 represents the regression result of enterprise R&D intensity (RD), and did2 represents the multiplication term of enterprise R&D intensity (RD) and DID terms. From the regression results, the regression coefficient of the first column intersection multiplication term (did1) is − 0.0003, passing the significance level of 1%. The regression coefficient of column 2 is 0.0162, which shows that the establishment of urban agglomeration can promote the improvement of enterprise innovation ability, institutional transaction cost and R&D investment intensity can play a regulating effect, and combined with the conclusion of the benchmark model, the integration effect of urban agglomeration on enterprise innovation can be reflected. This confirms the H1 hypothesis, that is, the implementation of urban agglomeration policies reduces the production costs of enterprises and promotes enterprise innovation through the integration effect.

**Table 6:** Inspection results of integration effect.

Variable	Cost	rd
did1	− 0.000380***	
	(− 3.15)	
did2		0.0162***
		(3.53)
DID	0.143*	− 0.159*
	(1.78)	(− 1.77)
Cost	0.000190***	
	(2.70)	
rd		0.0845***
		(13.91)
Size	0.447***	0.316***
	(11.53)	(8.70)
Age	− 0.781***	− 0.575***
	(− 4.76)	(− 5.04)
Lev	− 0.00827***	− 0.00577***
	(− 4.15)	(− 3.01)
Sub	− 0.0266	− 0.0329
	(− 1.01)	(− 1.31)
Growth	− 0.00503	0.000534
	(− 0.35)	(0.04)
Roa	0.811**	0.788**
	(2.38)	(2.02)
Cashflow	0.0582	0.0794
	(0.40)	(0.72)
_cons	2.599***	1.849***
	(4.30)	(3.35)
Induetry	Yes	Yes
Year	Yes	Yes
N	16,160	16,160
r2	0.450	0.490

### Spillover effect

The mechanism analysis shows that in the process of urban agglomeration development, the central city will have a siphon effect and diffusion effect on the surrounding cities. Under the condition that the siphon effect is prominent, the talents, capital and other production factors of surrounding cities are concentrated in the central cities, widening the regional development gap; while the spillover effect dominates, the central city drives the growth of labor and capital input in the surrounding cities, and the viscous knowledge is transmitted in the network platform of upstream and downstream enterprises forming a conductive production network and cooperation in the neighboring cities, promoting the innovation of enterprises in surrounding cities. Based on the proximity spillover and geographical first principle, this paper takes the central city as the base point, investigates the distance between the geographical distance as the main variable of overflow, and divides the distance between the city where the enterprise is located and the central city into 5 levels in units of 100 km, and the regression results are shown in Table 7. The results of Table 7 show that the impact of urban agglomeration policy on enterprise innovation within 100 km is positive but not significant. The impact on enterprises of 200–300 km is negative, but not significant. The impact on the innovation ability of enterprises at 100–200 km, 300–400 km and 400+ km was significantly positive, with the highest confidence in the 300–400 km range and slightly lower confidence in 400+ km. The possible reasons for this result are: firstly, the impact of urban agglomeration policy on the innovation of enterprises adjacent to the central city mainly depends on the spatial agglomeration effect of the original central city, and the spatial spillover effect mainly relies on the sub-central area within 200 km, and the siphon effect within 200–300 km is greater than the spillover effect, which also indicates that after the implementation of the urban agglomeration policy, the area within 300 km away from the central city is still the siphon and overflow mechanism of the original central city playing a major role. Secondly, in the areas greater than 300 km, the impact of urban agglomeration policy is the most significant, indicating that the implementation of urban agglomeration policy further promotes the process of urban and urban integration, and cities with a distance of more than 300 km are weakened by the polarization effect due to the distance from the central city, and the price of land, labor and other factors are lower, which can undertake the production capacity and technology transfer of more central cities, so it is reflected in the stronger impact of the diffusion effect, but the range above 400 km still reflects a certain geographical attenuation impact. This confirms the hypothesis H2, that is, the spillover effect of urban agglomeration policy on the promotion of enterprise innovation is affected by the attenuation of geographical distance, but the impact will gradually weaken with the deepening of the implementation of urban agglomeration policy.

**Table 7:** Spillover effect test results.

Variable	0–100 KM	100–200 KM	200–300 KM	300–400 KM	400+ KM
DID	0.0374	0.222*	− 0.184	0.615***	0.617*
	(0.26)	(1.83)	(− 0.81)	(3.18)	(1.87)
Size	0.529***	0.523***	0.530***	0.560***	0.539***
	(7.71)	(8.40)	(6.65)	(7.16)	(6.70)
Age	− 0.546**	− 0.540***	− 0.566**	− 0.395	− 0.425
	(− 2.57)	(− 2.87)	(− 2.24)	(− 1.62)	(− 1.60)
Lev	− 0.0114***	− 0.0101***	− 0.0123***	− 0.0123***	− 0.0103**
	(− 3.00)	(− 3.06)	(− 2.90)	(− 2.85)	(− 2.23)
Sub	− 0.0834*	− 0.0245	− 0.0707	− 0.0406	− 0.0355
	(− 1.86)	(− 0.60)	(− 1.42)	(− 0.81)	(− 0.69)
Growth	0.00233	0.0129	0.0254	0.0144	0.00939
	(0.08)	(0.55)	(0.84)	(0.48)	(0.29)
Roa	0.506	0.256	− 0.164	− 0.0234	0.169
	(0.78)	(0.42)	(− 0.22)	(− 0.03)	(0.22)
Cashflow	0.226	0.246	0.278	0.262	0.312
	(1.03)	(1.28)	(1.11)	(1.06)	(1.23)
_ cons	2.570***	1.475*	2.338**	1.132	1.152
	(2.69)	(1.67)	(2.24)	(1.08)	(1.08)
Industry	Yes	Yes	Yes	Yes	Yes
Year	Yes	Yes	Yes	Yes	Yes
N	5088	5896	3976	3912	3712
r2	0.442	0.457	0.445	0.470	0.454

## Heterogeneity analysis

After confirming the positive promotion effect of urban agglomeration policies on enterprise innovation and its impact on integration and spillover effects, this part continues to further explore the differentiated impact of urban agglomeration policies on enterprise innovation from the characteristics of heterogeneity of enterprises, industries and locations.

### Regression of property rights

In recent years, the research on the innovation ability of state-owned enterprises and non-state-owned enterprises is a hot issue of academic attention, believing that state-owned enterprises have strong financial strength, stable enterprise operation, strong anti-risk ability, can engage in innovation activities with high risk coefficient and long investment time, non-state-owned enterprises have a weak foundation, face a changing market environment at any time, and lack of ability to innovate activities that require a large amount of funds and talent investment for a long time. To a large extent, the difference in the nature of property rights reflect the difference in labor productivity of Chinese enterprises, so it is chosen to distinguish the heterogeneity of Chinese enterprises according to the nature of property rights.

Does the implementation of the urban agglomeration policy reduce the institutional transaction costs of enterprises through the integration effect, improve the market capacity and anti-risk ability of enterprises, and expand the scope of influence of spillover effects, which has an impact on enterprises with different property rights? Based on this, this paper further divides the sample into state-owned and non-state-owned enterprises according to the nature of property rights, and regresses the two samples separately, and the regression results are shown in Table 8. The regression results show that the regression coefficient of the sample of state-owned enterprises is 0.0776, which is not significant, and the regression coefficient of non-state-owned enterprises is 0.164, and through the significant level of 1%, it is revealed that the effect of urban agglomeration policy in promoting the innovation of non-state-owned enterprises is more obvious than that of state-owned enterprises. One possible reason for these findings is that state-owned enterprises generally have greater access to capital and labor resources, lower financing constraints, and stronger R&D investment capabilities. They also benefit from existing advantages in terms of transaction costs. As a result, the impact of urban agglomeration policies on state-owned enterprises is relatively small compared to non-state-owned enterprises. On the other hand, non-state-owned enterprises, particularly those located in remote cities and towns, can greatly benefit from urban agglomeration policies. These policies facilitate market integration, improve trading conditions, and enhance infrastructure development. As a result, the operating costs for non-state-owned enterprises are significantly reduced. Furthermore, these policies also stimulate market competition and encourage non-state-owned enterprises to increase their investment in research and development, thereby promoting their innovative development.

**Table 8:** Heterogeneity analysis results.

Variable	State-owned	Non-state-owned	Manufacturing	Service industry
DID	0.0776	0.164***	0.163*	− 0.110
	(0.63)	(3.13)	(1.65)	(− 0.74)
Size	0.322***	0.540***	0.626***	0.267***
	(5.83)	(27.03)	(11.77)	(4.78)
Age	− 0.695***	− 0.796***	− 0.802***	− 0.805***
	(− 3.05)	(− 15.43)	(− 5.35)	(− 3.57)
Lev	− 0.00376	− 0.0117***	− 0.00884***	− 0.00910***
	(− 1.13)	(− 10.79)	(− 3.30)	(− 2.66)
Sub	0.0536	− 0.0666***	− 0.0339	− 0.0457
	(1.34)	(− 4.27)	(− 0.95)	(− 1.26)
Growth	0.0156	− 0.0273*	− 0.0718***	0.00795
	(0.74)	(− 1.72)	(− 2.78)	(0.41)
Roa	1.067	0.598**	1.122**	0.468
	(1.47)	(2.10)	(2.13)	(0.60)
Cashflow	− 0.0290	0.165	0.256	− 0.0755
	(− 0.17)	(1.63)	(1.28)	(− 0.53)
_ cons	1.323	3.074***	2.222***	3.081***
	(1.35)	(11.14)	(3.00)	(3.35)
Industry	Yes	Yes	Yes	Yes
Year	Yes	Yes	Yes	Yes
N	7408	8752	9792	4448
r2	0.478	0.446	0.252	0.320

### Return by industry

From Weber’s industrial location theory to Krugman’s iceberg cost theory, the factors affecting the location of enterprises have been pointed out, and the implementation of urban agglomeration policies has greatly improved the internal location business environment, helped to attract investment from enterprises, formed a larger spatial agglomeration, and built an innovative enterprise foundation. Enterprises in different industries have different requirements for site selection, such as manufacturing industry is more sensitive to the price of land, labor, transportation and logistics costs, service industry has a greater preference for consumer market agglomeration, and whether different industries have differentiated responses to urban agglomeration policies is the objective basis for the further formulation of urban agglomeration planning, therefore this paper continues to distinguish manufacturing and service industries on the basis of enterprise heterogeneity, and conducts regression analysis. As can be seen from the regression results in Table 8, the regression coefficient of manufacturing industry is 0.163, which is significant at the significance level of 10%, and that of service industry is -0.110, which is not significant, indicating that the establishment of urban agglomeration has a positive effect on the innovation of manufacturing enterprises, but not on the innovation of service enterprises. The main reasons are: firstly, manufacturing enterprises are more sensitive to changes in transaction costs brought about by integration, and the spillover effect also provides more space for the location of enterprises, such as enterprises can locate factories in peripheral cities to reduce production costs, thereby investing the saved funds in R&D and innovation. Secondly, the service industry is divided into productive service industry and consumer service industry, with manufacturing and final consumers as the main service object, its innovation mainly comes from the innovation of formats and models, less innovation in invention patents, etc., so it is less affected by the reduction of transaction costs, and at the same time, the integrated construction of urban agglomerations changes the functions of cities and towns, which will restructure the service industry, and also have a certain lagging impact on the innovation of the service industry. According to the sectoral regression study, heterogeneous industries have different responses to urban agglomeration policies, manufacturing innovation is positively affected, and service innovation is inhibited to a certain extent.

### Regression by urban agglomeration

Among the 11 state-level urban agglomerations that have been approved, there are great differences in geographical location, industrial base, economic development level, integration degree and other aspects of the urban agglomerations. Existing studies show that different urban agglomerations have different driving effects on regional regions due to different structures, and have different effects on enterprise innovation. In order to further investigate whether there are differences in the impact of different urban agglomerations on the innovation ability of enterprises, this paper selects four major urban agglomerations of Beijing-Tianjin-Hebei, Yangtze River Delta, Pearl River Delta and Chengdu-Chongqing for sub-sample breakpoint regression, and the regression results are shown in Table 9. Table 9 shows that among the four major urban agglomerations, the sample regression coefficient of Beijing-Tianjin-Hebei urban agglomeration is 0.464, which is significant at 5%. The regression coefficient of the Yangtze River Delta urban agglomeration is 0.410, which is significant at 1% level. The regression coefficient of the Pearl River Delta urban agglomeration is 0.483, which is significant at the significance level of 1%. The sample regression coefficient of Chengdu-Chongqing urban agglomeration is 1.130, which is significant at 1% significance level, indicating that the four urban agglomerations have a certain promoting effect on enterprise innovation, but the effect of different urban agglomerations is different. The reasons are: firstly, the Yangtze River Delta urban agglomeration accounts for 2.2% of China’s regional area, is the area of the four major urban agglomerations. Secondly, Beijing-Tianjin-Hebei urban agglomeration is one of the most dynamic and innovative regions in China’s regional economic development, the Yangtze River Delta urban agglomeration forms a diamond-like spatial pattern in the shape of “Z”, with multi-center driving characteristics. The Pearl River Delta city cluster is the main area of China’s participation in economic globalization and the national scientific and technological innovation and technology research and development base, highlighting the multi-center driving pattern of cities on both sides of the Pearl River Estuary as the center and other cities as the nodes in the spatial pattern, therefore the implementation of the city cluster policy in the Yangtze River Delta and the Pearl River Delta can further affect the innovation of enterprises through the collaborative network, resulting in a good policy spillover effect. Thirdly, the positioning of the Beijing-Tianjin-Hebei urban agglomeration is different from that of the Yangtze River Delta and the Pearl River Delta, and the Beijing-Tianjin-Hebei urban agglomeration is committed to building a new capital economic circle, promoting the innovation of regional development systems and mechanisms, exploring and improving the layout of urban agglomerations and an effective path for ecological civilization construction, and innovation links more rely on the radial development of central cities, so the institutional innovation represented by urban agglomeration policies has obvious innovation effects on enterprises. Fourthly, the Chengdu-Chongqing urban agglomeration is positioned as an important platform for the opening-up of western China centered on Chongqing and Chengdu, and is an important economic center, science and technology innovation center and new highland of reform and opening-up in the west.

**Table 9:** Regression results of four urban agglomerations.

Variable	Beijing tianjin hebei	Changjiang delta	Pearl river delta	Chengdu chongqing
D	0.464**	0.410***	0.483***	1.130***
	(2.12)	(3.88)	(2.85)	(3.84)
Size	0.0677	0.0691**	0.0843	0.172
	(1.28)	(2.16)	(1.32)	(1.51)
Age	0.941**	0.726***	0.699**	− 0.879
	(2.10)	(3.17)	(2.22)	(− 1.39)
Lev	0.000651	0.00121	0.00160	0.00687
	(0.26)	(0.86)	(0.90)	(1.49)
Sub	0.0297	0.00273	− 0.000995	− 0.0761*
	(0.92)	(0.20)	(− 0.04)	(− 1.88)
Growth	− 0.00554	0.00170	− 0.0109	− 0.00404
	(− 0.59)	(0.26)	(− 1.06)	(− 0.35)
Roa	− 0.594	0.120	− 0.287	0.521
	(− 1.21)	(0.55)	(− 1.07)	(0.76)
Cashflow	− 0.0580	− 0.00331	0.0107	− 0.300**
	(− 0.44)	(− 0.07)	(0.13)	(− 2.39)
_ cons	− 0.852	0.430	0.598	4.402**
	(− 0.72)	(0.66)	(0.49)	(2.40)
Industry	Yes	Yes	Yes	Yes
Urban	Yes	Yes	Yes	Yes
Individual	Yes	Yes	Yes	Yes
Year	Yes	Yes	Yes	Yes
N	2016	4800	2096	832
r2	0.322	0.281	0.303	0.198

## Conclusions and policy recommendations

In order to quantitatively explore the possible effects of urban agglomeration policies on the innovation of micro market players, this paper introduces the exogenous variable of urban agglomeration policies, and focuses on the mechanism derivation and quantitative analysis from the aspects of integration effect and spillover effect, in an attempt to provide objective basis for regional policy planning and promote high-quality regional development. The conclusions are as follows:

Firstly, regarding the establishment of urban agglomeration as a quasi-natural experiment and using the multi-period double difference method to study whether the implementation of urban agglomeration policies can promote the improvement of enterprises’ innovation ability, and conduct a parallel trend test. Test endogenous problems by eliminating central cities. In addition, conduct a robustness test by replacing dependent variables and counterfactual simulation methods, objectively revealing that urban agglomeration policies can promote enterprise innovation. The regional policy is of great significance for constructing a high-quality development platform for micro entities.

Secondly, the integration effect is characterized by the transaction cost measurement as the core independent variable. The regulatory effect test on this shows that the implementation of the urban agglomeration policy can improve the integration level of the market, system and infrastructure, break down the institutional barriers and barriers to trade, reduce the transaction cost of enterprises, promote enterprises to increase their R&D investment, and promote enterprise innovation and development.

Thirdly, the study on spillover effect based on the geographical distance structure sub sample model shows that the siphon and spillover mechanism of the central cities of urban agglomeration still plays an effective role. The promotion of spillover effect on enterprise innovation is affected by the attenuation of geographical distance, but the influence of geographical attenuation gradually weakens under the intervention of urban agglomeration policies, and the spillover scope of urban agglomeration is expanded. It shows that the urban agglomeration policy has a certain regulatory effect on the self-mechanism of the formation of the central city, and can drive the innovative development of the peripheral micro market subjects.

Fourthly, the further research on the heterogeneity of sub samples from the perspective of enterprises, industries and locations reveals that the role of urban agglomeration policies is different in macro, medium and micro effects. At the macro level, urban agglomeration with a multi-center structure has a significant impact on enterprise innovation. At the micro level, the positive impact of urban agglomeration policies on the manufacturing industry innovation is much higher than that of the service industry. At the micro level, the positive impact of urban agglomeration policies on the innovation of non-state-owned enterprises is greater than that of state-owned enterprises, so the policy formulation of urban agglomeration should also be different.

Based on the above research conclusions, this paper proposes the following countermeasures and suggestions:

Firstly, continue to promote the policy planning of urban agglomeration, improve the inter provincial and inter-city consultation and coordination mechanisms, and enhance policy coordination. On the one hand, establish a long-term and effective policy consultation mechanism among cities within the urban agglomeration, accelerate the integrated development of urban agglomeration, further remove administrative barriers and institutional barriers between cities, improve administrative efficiency, and optimize the business environment. On the other hand, break market segmentation and local protectionism, constantly improve laws and regulations, improve fiscal and taxation policies, accelerate the formulation of unified market system rules, abolish policies that discriminate against foreign enterprises, negotiate and unify market supervision rules, standards and procedures, accelerate the construction of a unified market with standardized, open, fair and benign competition, and form a market environment for fair and benign competition between local and foreign enterprises. This will reduce the transaction cost of enterprises and increase the proportion of capital investment in R&D.

Secondly, promote the coordination between the policy planning of urban agglomeration and the siphon and spillover effects of cities within the agglomeration, and adjust the impact of the self-mechanism of urban agglomeration. According to the borrowing scale and gathering shadow with the central city as the core formed spontaneously by the urban agglomeration, fully consider the economic development level and competitive strength of the central city and other cities, identify the economic hinterland that can be selected in combination with the resource endowment and industrial foundation, give play to the advantage cooperation among cities, create policy conditions to expand the spillover scope of the central city, and promote the innovative development of enterprises within the scope.

Thirdly, promote the adaptation of the policy planning of urban agglomeration to the differentiation, phasing and continuity of the functional positioning of urban agglomeration, and promote the formation of the multi center innovation structure and network of urban agglomeration. According to the structural characteristics of different urban agglomerations, dynamic adaptive policies should be formulated according to local conditions to better exert the spillover effect of urban agglomeration policies. Promote the growth of competitive multi center cities through differentiation policies, select advantageous clusters to lead the development of regional industries, establish a sound intermediary market, and further build the production network within the city cluster through the proximity of the cluster network, thus promoting the development of enterprise innovation networks and improving the innovation efficiency of regional enterprises.

## Data Availability

The datasets generated and/or analyzed during the current study are available from the corresponding author upon reasonable request. Data access will be granted after approval of a data sharing agreement, which will detail the terms and conditions for use of the data. The materials used in this study are available upon request from the corresponding author, subject to any legal or ethical constraints. Any requests for data or materials should be directed to the corresponding author for consideration. We believe that this approach will balance the need for transparency and data accessibility with the importance of respecting the privacy and confidentiality of the participants in our study.
